# Klotho in Cancer: Potential Diagnostic and Prognostic Applications

**DOI:** 10.3390/diagnostics13213357

**Published:** 2023-10-31

**Authors:** Jucileide Mota, Alice Marques Moreira Lima, Jhessica I. S. Gomes, Marcelo Souza de Andrade, Haissa O. Brito, Melaine M. A. Lawall Silva, Ana I. Faustino-Rocha, Paula A. Oliveira, Fernanda F. Lopes, Rui M. Gil da Costa

**Affiliations:** 1 Post-Graduate Programme in Adult Health (PPGSAD), Federal University of Maranhão, São Luís 65085-580, Brazil; 2 Health Sciences Center, State University of the Tocantins Region of Maranhão (UEMASUL), Imperatriz 6591-480, Brazil; 3 Morphology Department, Federal University of Maranhão, São Luís 65085-580, Brazil; 4 Centre for the Research and Technology of Agro-Environmental and Biological Sciences (CITAB), University of Trás-os-Montes and Alto Douro, 5000-801 Vila Real, Portugal; 5 Inov4Agro—Institute for Innovation, Capacity Building and Sustainability of Agri-Food Production, University of Trás-os-Montes and Alto Douro, 5000-801 Vila Real, Portugal; 6 Laboratory for Process Engineering, Environment, Biotechnology and Energy (LEPABE), Faculty of Engineering, University of Porto, 4200-465 Porto, Portugal; 7 Associate Laboratory in Chemical Engineering, Faculty of Engineering (ALiCE), University of Porto, 4200-465 Porto, Portugal; 8 Molecular Oncology and Viral Pathology Group, Portuguese Oncology Institute of Porto (IPO Porto), 4200-072 Porto, Portugal; 9 Health Research Network, Research Center of Portuguese Oncology Institute of Porto (CIIPOP/RISE@CIIPOP), 4200-072 Porto, Portugal

**Keywords:** liquid biopsy, cancer, klotho, prognosis, diagnosis

## Abstract

Klotho proteins, αKlotho, βKlotho, and γKlotho, exert tumor-suppressive activities via the fibroblast growth factor receptors and multiple cell-signaling pathways. There is a growing interest in Klotho proteins as potential diagnostic and prognostic biomarkers for multiple diseases. However, recent advances regarding their roles and potential applications in cancer remain disperse and require an integrated analysis. The present review analyzed research articles published between 2012 and 2022 in the Cochrane and Scopus scientific databases to study the role of Klotho in cancer and their potential as tools for diagnosing specific cancer types, predicting tumor aggressiveness and prognosis. Twenty-six articles were selected, dealing with acute myeloid leukemia and with bladder, breast, colorectal, esophageal, gastric, hepatocellular, ovarian, pancreatic, prostatic, pulmonary, renal, and thyroid cancers. αKlotho was consistently associated with improved prognosis and may be useful in estimating patient survival. A single study reported the use of soluble αKlotho levels in blood serum as a tool to aid the diagnosis of esophageal cancer. γKlotho was associated with increased aggressiveness of bladder, breast, and prostate cancer, and βKlotho showed mixed results. Further clinical development of Klotho-based assays will require careful identification of specific tumor subtypes where Klotho proteins may be most valuable as diagnostic or prognostic tools.

## 1. Introduction

The Klotho proteins, alpha(α)Klotho [1,2] and beta(β)Klotho [3], are encoded by the *KLA* and *KLB* genes located in chromosomes 4 and 13, respectively. αKlotho was originally identified in mice and elicited great interest due to its anti-aging properties [1]. It is expressed in a variety of tissues and is in the cell membrane as a type I single-pass 135 kDa protein containing an N-terminal sequence, two extracellular domains (designated KL1 and KL2) with glycosidase activity, a transmembrane helix, and an intracellular domain consisting of only 10 amino acids [2].

The αKlotho protein is also present in blood as a secreted protein generated by alternative mRNA splicing containing the KL1 domain only [1] and as a soluble protein that may contain KL1 alone or both the KL1 and Kl2 extracellular domains [4]. Cleavage of the αKlotho extracellular domains is mediated by disintegrin and metalloproteinase domain-containing (ADAM) proteins ADAM10 and ADAM17 [4]. The βKlotho protein shares structural similarities with αKlotho and is also located in the cell’s plasma membrane [3,5], and soluble βKlotho has also been reported [6]. Another membrane-bound glycosidase-like protein, designated Klotho-lactase phlorizin hydrolase, was first identified in mice and is encoded by the *LCTL* gene on chromosome 15 in humans [7]. The functions of this protein, also referred to as γKlotho, are less clear than those of αKlotho and βKlotho.

αKlotho binds to FGR receptors, acting as a co-receptor for FGF23 and playing a key role in the renal regulation of phosphate levels [8,9]. βKlotho acts as a co-receptor for fibroblast growth factors 19 and 21 (FGF19 and FGF21) by forming binary complexes with FGFR4 and FGFR1c, respectively [10,11,12]. The binding of βKlotho with FGFR1c in adipose tissue or FGFR4 in the liver and with endocrine ligands FGF21 and FGF19 triggers multiple intracellular responses, as previously reviewed [5]. Canonically, the binding of FGF21 to the βKlotho-FGFR1c complex activates ERK1/2 downstream signaling and regulates the synthesis of biliary acids in hepatocytes, while FGF19 binds to βKlotho-FGFR4 complexes to downregulate Cyp17a1, also regulating hepatic bile production [11,12,13,14].

**Figure 1:**
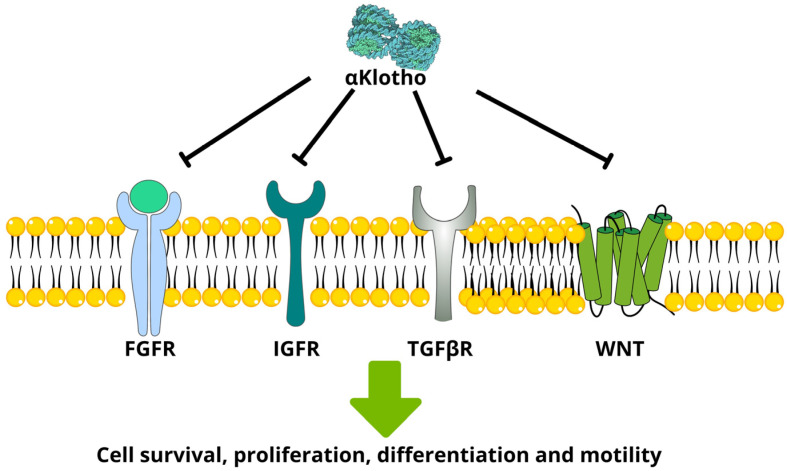
αKlotho downregulates signaling mediated by multiple cell membrane receptors, contributing to its anti-cancer effects.

Loss of αKlotho has been consistently linked with chronic kidney disease and phosphate metabolism dysfunction [15,16]. αKlotho downregulation was also associated with pleiotropic effects involved in aging [1,5] and is proposed to act as a tumor suppressor, as recently reviewed [17]. Interestingly, βKlotho has been associated with both tumorigenic and tumor-suppressive effects in different types of cancer, suggesting a more complex scenario with multiple context-specific activities [18,19,20]. γKlotho expression has also been studied in multiple types of cancer [21,22]. In cancer, Klotho proteins have been shown to interact with multiple cellular signaling pathways, enhancing or blocking carcinogenesis, as previously reviewed [17,23]. As well as interacting with FGF to activate FGFR, αKlotho (Figure 1) was initially found to downregulate signaling via insulin-like growth factor 1 receptor (IGF-1R), and this may contribute to its effects against some types of cancer [24,25]. βKlotho enhances pro-tumorigenic functions of FGFR in multiple types of cancer [26,27]. The phosphatidylinositol-3-kinase (PI3K) pathway is triggered by multiple membrane-bound receptors and mediates cell proliferation, growth, and survival and is also inhibited by αKlotho [28]. The WNT-β-catenin pathway is activated in multiple cancers where it modulates cell differentiation, survival, and mobility [29]. αKlotho’s ability to block this pathway contributes to its anti-tumor properties [30]. Transforming growth factor beta (TGFβ) is also able to modulate cell differentiation and mobility, namely inducing epithelial-to-mesenchymal transition [31], and αKlotho can block those effects [32]. The signaling pathways modulated by γKlotho are less studied, but Hori et al. (2016) implicated this protein in epithelial-to-mesenchymal transition in bladder cancer.

Accumulating data suggests that the tissue expression of Klotho proteins and, especially, the detection and quantitation of their soluble forms in body fluids like blood serum may be useful for establishing the diagnosis and prognosis of some types of cancer [6,33,34]. The present review aims to analyze scientific data regarding the role of Klotho proteins in cancer and to retrieve information regarding their potential use as diagnostic and prognostic biomarkers.

## 2. Materials and Methods

**Figure 2:**
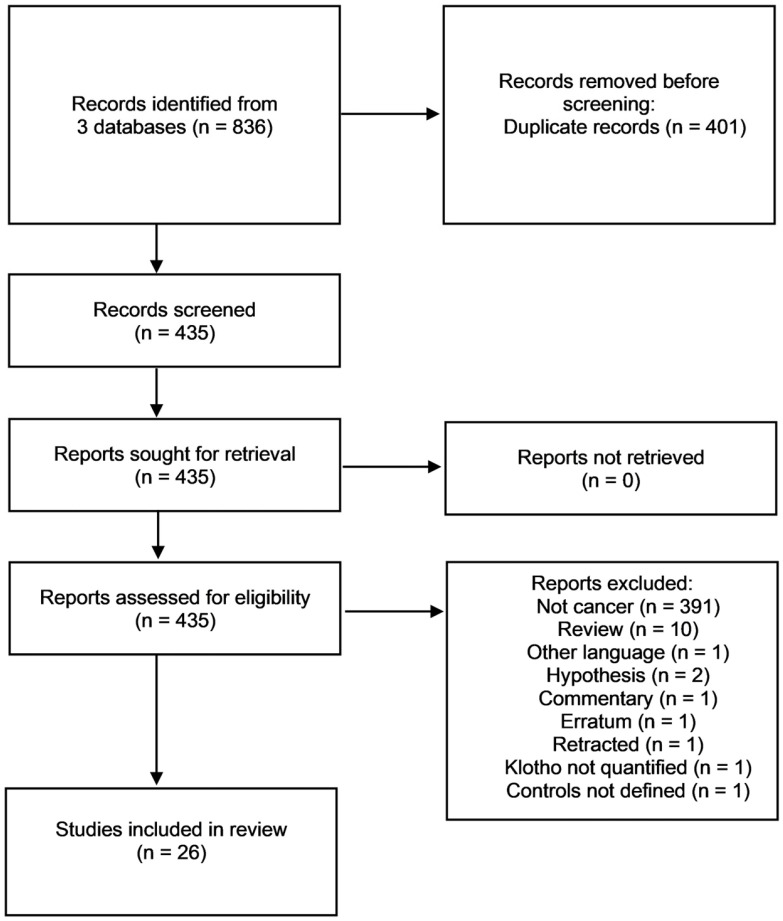
Selection of articles from the PubMed, Scielo, and ScienceDirect databases and resulting publications for analysis.

The review was performed on three standard databases on biomedicine: PubMed, Scielo, and ScienceDirect, accessed in April 2023, including scientific papers published between 2012 and December 2022. The keywords “cancer AND Klotho” were applied. The following inclusion criteria were established concerning the type of study (case series and case–control studies in humans; experimental in vitro and in vivo studies) and outcomes (effects of Klotho gene products in cancer). Exclusion criteria were lack of clear definition of cancer type or controls, lack of Klotho gene product quantification, case reports, review articles, commentaries, hypothesis and meta-analyses, and languages other than English. The abstracts and, when necessary, the materials and methods were analyzed to apply inclusion and exclusion criteria (Figure 2).

## 3. Results

Most publications were excluded due to duplication between databases or by applying exclusion criteria. Many articles have dealt with other pathologies where Klotho proteins are thought to play significant roles, most prominently in renal diseases. Overall, after applying inclusion and exclusion criteria, 26 articles were selected for further analysis (Table 1). Most studies used in vitro and/or clinical observational approaches, with only 7 articles using in vivo studies with animal models. Clinical observational studies often described the expression of Klotho genes at the RNA and/or protein levels and provided correlations between these markers’ expression levels and relevant clinical parameters. Caseloads varied between 36 and 313 patients. Remarkably, none of the clinical studies adopted an interventional approach, and most consisted of retrospective cohort studies, while one article included a case–control study. In vitro studies provided insights into the regulation of Klotho protein’s expression and its effects on cancer cells. Among the 26 selected articles, 21 dealt with αKlotho, 5 with βKlotho and only 3 with γKlotho, with one article studying α and βKlotho and another studying all the three proteins.

### 3.1. αKlotho

The main findings of the 21 articles addressing αKlotho in cancer are summarized in Table 2. Four studies were focused on colorectal cancer [46,48,50,53], another three on lung cancer [35,42,45], two on hepatocellular carcinoma [30,36], two on ovarian cancer [38,43], two on renal cell carcinoma [28,37], and two on gastric cancer [49,52]. Prostate cancer [27], acute myeloid leukemia [39], thyroid cancer [40], esophageal cancer [41], breast cancer [21], and pancreatic cancer [51] were each studied by a single article.

#### 3.1.1. Clinicopathological Characteristics

αKlotho was generally found to act as a tumor suppressor, and its downregulation was consistently associated with aggressive tumor phenotypes and worse prognosis. In prostate cancer, αKlotho protein expression was detected in 50% of primary and 90% of metastatic samples [27]. In lung cancer, αKlotho was detected in most samples, but its expression pattern seems to be subtype-specific and requires further studies [40,45]. In hepatocellular carcinoma, αKlotho tissue expression is downregulated in tumor versus adjacent tissues and inversely correlates with tumor size, TNM stage, and nuclear grade [36]. Similar findings were obtained when studying renal cell carcinoma [30,39]. In breast [21], esophageal [41] and ovarian [38,43] cancer, αKlotho expression is downregulated compared with normal tissues.

#### 3.1.2. Diagnosis

Soluble αKlotho can be quantified in blood serum using ELISA, and αKlotho levels were also suggested to have diagnostic value for esophageal cancer [41].

#### 3.1.3. Survival and Treatment Response

The quantitation of αKlotho expression levels on tumor tissues using immunohistochemistry (IHC) was of prognostic significance in colorectal, esophageal, hepatocellular, lung, and ovarian cancer [36,41,43,45,46]. *KLA* promoter methylation and mRNA expression levels by quantitative real-time PCR (qRT-PCR) were also reported to have prognostic value in hepatocellular carcinoma and pancreatic cancer [36,51]. Reduced αKlotho serum levels were associated with reduced cancer-specific survival and progression-free survival among renal cell carcinoma patients [37]. Interestingly, reduced αKlotho levels were also suggested to promote cytarabine resistance in acute myeloid leukemia cells [39].

### 3.2. βKlotho

The 5 articles focused on βKlotho are addressed in Table 3, which summarizes their main findings. Two articles dealt with hepatocellular carcinoma [26,54], while prostate cancer [27], breast cancer [21], and pancreatic adenocarcinoma [44] were studied in one article each.

#### 3.2.1. Clinicopathological Characteristics

In hepatocellular carcinoma, βKlotho was proposed to mediate tumor aggressiveness via FGFR signaling [26,54]. Conversely, in breast and pancreatic cancers, βKlotho was proposed to act as a tumor suppressor [21,44]. In prostate cancer, βKlotho protein expression was detected in a majority of primary and metastatic lesions [27].

#### 3.2.2. Survival and Treatment Response

Interestingly, one study on hepatocellular carcinoma [26] showed that a >2-fold increase in *KLB* gene expression correlates with the development of multiple versus single lesions. A pre-clinical study [55] suggested that βKlotho mediates FGF9 pro-survival functions in hepatocellular carcinoma via FGFR3 and FGFR4 and may be useful in selecting patients who could benefit from anti-FGFR therapies. A similar scenario was suggested by a single study focused on prostate cancer [27].

### 3.3. γKlotho

γKlotho was studied in three articles, summarized in Table 4. Breast [21], prostate [47], and bladder cancers [22] were studied in one article each. All three articles found that higher γKlotho expression is associated with cancer aggressiveness and poor prognosis, suggesting that γKlotho levels assessed at the mRNA or the protein level may be useful to predict patient survival and response to therapy.

#### 3.3.1. Clinicopathological Characteristics

Triple-negative breast cancer is an aggressive breast cancer subtype that poses a significant therapeutic challenge [56]. *LCTL* gene expression was found to be upregulated in triple-negative breast cancer samples, and expression levels correlated with increased cell proliferation, histological grade, and TNM stage [21]. Bladder cancer includes muscle-invasive and non-muscle-invasive forms [57] with distinct biological behavior. Higher γKlotho protein expression was observed in muscle-invasive versus non-muscle-invasive lesions [22].

#### 3.3.2. Survival and Treatment Response

In triple-negative breast cancer, *LCTL* gene expression levels correlated with reduced progression-free survival [21]. Castration-resistant prostate cancer is another challenging malignancy with heterogeneous morphological and molecular phenotypes [55,58]. High γKlotho expression levels, as demonstrated by IHC, were shown to correlate with reduced overall survival and poor response to docetaxel in patients and in a mouse xenograft model [40]. In non-muscle-invasive bladder cancer, γKlotho protein levels were shown to correlate with reduced progression-free survival [22].

## 4. Discussion

The three Klotho proteins have complex roles in different types of cancer. The role of γKlotho is less well defined than that of its related Klotho proteins, partially because of its unusual molecular structure and because it was discovered more recently. The present review organized data from scientific articles published between 2012 and 2022 regarding the roles of Klotho proteins in cancer and their potential use as diagnostic and prognostic tools.

The role of all three proteins was studied in prostate cancer. This is a highly prevalent disease in middle-aged to older men that usually develops as an androgen-dependent adenocarcinoma but may progress to an androgen-independent castration-resistant phenotype and small-cell neoplasia, often displaying neuroendocrine markers, which are associated with poor patient prognosis [55]. αKlotho and βKlotho expression was detected in prostate cancer cell lines representing prostate adenocarcinoma and small-cell carcinoma, as well as in tumor tissues from primary tumors and metastasis, where they seem to mediate FGFR signaling [27]. It was further suggested that IHC tests for detecting αKlotho and βKlotho in tumor tissue may be of use to predict response to anti-FGFR therapies [27]. γKlotho expression in castration-resistant prostate cancer was associated with reduced survival and resistance to docetaxel [47], which is used as chemotherapy for such advanced cases [59]. Taken together, these results suggest that the immuno-expression patterns of Klotho proteins on prostate cancer tissues may be a valuable tool for tailoring treatment regimens for specific patients.

Lung cancer is also a common and aggressive malignancy, which includes multiple subtypes with distinct biological behavior [60]. Loss of αKlotho expression was consistently associated with increased tumor aggressiveness in three studies using in vitro and in vivo models [35] and clinical observational studies of neuroendocrine tumors [45], early centrally located cancers, and squamous cell carcinomas [42]. The observation that αKlotho may predict survival in patients with large cell neuroendocrine lung cancer is of particular interest, as it suggests that this marker has prognostic value in this specific lung cancer subtype [45]. Additionally, limited in vivo and in vitro data suggest that αKlotho downregulation may predict resistance to cisplatin-based chemotherapy [35], but additional studies are required to confirm this hypothesis.

Hepatocellular carcinoma is the most common type of liver cancer [61]. Although αKlotho was reported to act as a tumor suppressor [30,36], βKlotho showed oncogenic activity via enhanced FGFR signaling [26,54]. Importantly, αKlotho gene promoter methylation and protein expression may be of use as prognostic markers to estimate patient survival [36], while βKlotho may be a useful marker to predict response to anti-FGFR therapies [26].

In renal cell carcinoma, αKlotho downregulation was also reported to act as a tumor suppressor, and its loss was associated with tumor aggressiveness [28,42]. Of particular interest is the use of ELISA tests to detect soluble αKlotho in blood serum samples, as reduced levels of this protein were significantly associated with patients with the clear cell subtype of RCC [37]. These findings suggest that such tests may be used in liquid biopsies to help establish the prognosis of specific RCC patient subgroups.

Ovarian cancer is a frequent malignancy in women [62], and αKlotho was reported to act as a tumor suppressor in this type of cancer using experimental and clinical approaches [38,44]. Importantly, one study suggested that reduced αKlotho immuno-expression in cancer tissues may be useful as a prognostic marker to predict poor patient survival [44]. The same study reported that αKlotho was associated with higher interleukin-6 (IL-6) circulating levels. IL-6 is a pro-inflammatory cytokine that mediates some paraneoplastic syndromes like cancer cachexia [63], so it is interesting to speculate that αKlotho expression levels may also be used to predict the development of such syndromes.

In acute myeloid leukemia, loss of αKlotho was reported to be associated with cytarabine resistance in vitro, suggesting its possible use as a tool to design tailored therapies for leukemia patients [39]. Additional studies are needed to test this hypothesis, as cytarabine remains an important drug for treating this type of leukemia [64].

Breast cancer is highly prevalent in women and is often life-threatening [56]. In one study, αKlotho and βKlotho were downregulated in tumor tissue versus adjacent tissue, suggesting they act as tumor suppressors [21]. Conversely, higher γKlotho (*LCTL*) gene expression levels using qRT-PCR were found in cancer versus adjacent tissue, specifically in the aggressive triple-negative cancer subtype [21,65], suggesting it is associated with tumor aggressiveness. Interestingly, it was suggested that qRT-PCR for *LCTL* may be useful as a prognostic marker to estimate patient survival in patients with triple-negative breast cancer [21].

In papillary thyroid cancer, a single study [40] reported that αKlotho was able to reduce cell proliferation and induce apoptosis in vitro. The potential use of this protein for diagnostic and prognostic purposes in thyroid cancer remains to be determined.

In esophageal cancer, an interesting study [41] reported that the levels of soluble αKlotho in blood serum as detected by ELISA were higher in patients versus healthy controls. A cut-off value was estimated that allowed researchers to distinguish between patients and controls with approximately 81% sensitivity and specificity. Interestingly, in tissue samples, αKlotho was expressed at higher levels in adjacent versus tumor samples, and αKlotho downregulation correlated with increased tumor aggressiveness and reduced patient survival. These data highlight the potential of αKlotho as a marker in liquid biopsies for the diagnosis of esophageal cancer, while tissue levels may have prognostic significance.

Colorectal cancer is highly prevalent in multiple world regions, and large bowel carcinogenesis is associated with chronic inflammation [66]. In this type of cancer, 4 studies consistently reported that αKlotho acts as a tumor suppressor [46,48,50,53]. In vitro tests revealed new regulatory pathways that control αKlotho expression via FL-1 [48] and support the pro-apoptotic role of αKlotho via TRAIL [50]. Interestingly, one study described how αKlotho downregulation promotes a senescence-associated secretory phenotype in mesenchymal cells that may contribute to tumorigenesis via the nuclear factor kappa-light-chain-enhancer of activated B cells (NFκB) signaling pathway [46]. This is a pivotal mediator of inflammation and tissue repair, but also of carcinogenesis in specific settings. Chronic inflammation is a key player in colon cancer, and the secretion of NFκB-controlled C-C motif chemokine ligand 2 (CCL2) by senescent stromal cells was proposed to promote carcinogenesis of the colon. αKlotho abrogated CCL2 signaling and was associated with improved patient survival, suggesting it may be of use as a prognostic marker.

Two in vitro studies addressed the role of αKlotho in gastric cancer, further associating αKlotho downregulation with aggressive cancer phenotypes [49,52]. SOX17 and an epigenetic pathway involving circular RNA ITCH and miR-199-5p were shown to regulate αKlotho expression in gastric cancer cells. Although these findings support the role of αKlotho as a tumor suppressor, further developments are needed to explore its potential role as a diagnostic or prognostic marker in gastric cancer.

A single study addressed the role of αKlotho in pancreatic adenocarcinoma and concluded that *KLA* gene expression levels and promoter methylation may have prognostic value, as increased *KLA* promoter methylation and decreased mRNA expression levels were associated with lower patient survival [51]. This was further supported by tests in three complementary mouse models, where αKlotho decreased cancer growth and improved survival. Another study using expression data from the GEO database also suggested that *KLB* upregulation is associated with improved survival in pancreatic cancer patients [46]. Taken together, these data provide evidence to support the further development of Klotho as a prognostic marker in pancreatic adenocarcinoma.

Urothelial carcinoma of the urinary bladder is a common malignancy that includes highly aggressive forms that invade the bladder’s muscular layer and non-muscle-invasive forms associated with local recurrence [67]. One study reported that γKlotho expression was observed in both muscle-invasive and non-muscle-invasive bladder cancer using IHC and that expression levels were associated with poor overall survival among patients with non-muscle-invasive cancer [22].

## 5. Conclusions

Overall, the datasets published between 2012 and 2022 provide evidence supporting the development of Klotho genes and their mRNA and protein products as potential prognostic markers in multiple types of cancer, especially in the prediction of patient survival. Although αKlotho was consistently associated with improved patient prognosis, γKlotho was associated with increased cancer aggressiveness, and βKlotho showed mixed results. It is critical to accurately identify specific tumor subtypes where Klotho is of interest (muscle-invasive versus non-muscle-invasive urothelial carcinoma) to take the most advantage of its potential. The use of Klotho levels as diagnostic markers was less frequently observed in the literature, although one study provided detailed data regarding soluble αKlotho levels in blood serum and the diagnosis of esophageal cancer. However, most studies still did not present such detailed results, and the clinical use of Klotho will require additional development.

## Figures and Tables

**Table 1:** Characteristics of the 26 articles included in the review.

Reference	Year	Type of Cancer	In Vitro	In Vivo	Number of Patients	Clinical (Observational)
[26]	2012	Hepatocellular carcinoma	x		56	Retrospective cohort
[27]	2013	Prostate cancer	x		136	Retrospective cohort
[35]	2013	Lung cancer	x	x		-
[36]	2013	Hepatocellular carcinoma	x		64	Retrospective cohort
[28]	2013	Renal cell carcinoma	x		125	Retrospective cohort
[37]	2015	Renal cell carcinoma			160	Retrospective cohort
[38]	2015	Ovarian cancer	x		265	Retrospective cohort
[39]	2015	Acute myeloid leukemia	x		109	Retrospective cohort
[30]	2015	Hepatocellular carcinoma	x			-
[21]	2015	Breast cancer	x		68	Retrospective cohort
[40]	2016	Thyroid cancer	x			-
[41]	2016	Esophageal cancer	x		160	Retrospective case–control
[42]	2017	Pulmonary squamous cell carcinoma	x		40	Retrospective cohort
[43]	2017	Ovarian cancer	x	x	198	Retrospective cohort
[44]	2018	Pancreatic adenocarcinoma	x		313	Retrospective cohort
[22]	2018	Bladder cancer	x	x	205	Retrospective cohort
[45]	2019	Large cell neuroendocrine lung cancer				Retrospective cohort
[46]	2019	Colorectal cancer	x	x	143	Retrospective cohort
[47]	2020	Prostate cancer		x	36	Retrospective cohort
[48]	2020	Colorectal cancer	x			-
[49]	2020	Gastric adenocarcinoma	x			-
[50]	2021	Colorectal cancer	x			-
[51]	2021	Pancreatic cancer		x	178	Retrospective cohort
[52]	2021	Gastric cancer	x		94	Retrospective cohort
[53]	2022	Colorectal cancer	x			-
[54]	2022	Hepatocellular carcinoma	x	x		-

x denotes that in vivo and/or in vitro experiments were performed for each article.

**Table 2:** Studies dealing with αKlotho.

Cancer Type	Reference	Type of Sample	Main Findings	Potential Applications
Prostate cancer	[27]	Frozen and FFPE cancer tissues. PC3, DU145, VCaP, LNCaP cancer cell lines, PNT1a normal prostate cells	*KLA* gene expression detected in all cell lines by qRT-PCR and FGF19 stimulates PCa cells in vitro. αKlotho detected by IHC in 50% primary and 90% metastatic PCa samples	Screening of patients who may benefit from anti-FGFR therapies and may be using IHC on tumor tissues
Lung cancer	[35]	A549 and H460 tumor cells and xenografts	αKlotho downregulation promotes cisplatin resistance in vitro and in vivo	
	[42]	FFPE cancer tissues (centrally located early lung cancer and SCC), A549, and SQ5 tumor cell lines	αKlotho expressed in 100% centrally located early lung cancer samples but only in 13% SCC using IHC. Inhibited N-cadherin expression in vitro	
	[45]	FFPE cancer tissues (large cell neuroendocrine lung cancer)	αKlotho expressed in ¾ patients and associated with survival	Tissue expression may predict prognosis (survival)
Hepatocellular carcinoma	[36]	Frozen and FFPE tumor and adjacent tissues. HRPG2, BEL-7402, SMMC-7721, HL7702, HUH-7, MHCC-97-H cancer cell lines and L-02 hepatocytes	αKlotho is downregulated at mRNA and protein levels in HCC versus adjacent tissue; promoter methylation and reduced protein expression correlate with reduced survival	αKlotho promoter methylation and protein expression may predict prognosis (survival)
	[30]	HepG2 and SMMC-7721 cancer cell lines, L-02 hepatocytes	Recombinant αKlotho downregulates Wnt/β-catenin signaling, suppressing proliferation and inducing apoptosis	
Renal cell carcinoma	[28]	786-O, OS-RC-2, ACHN, Caki-1 and Renca cancer cell lines. Tumor tissue	αKlotho tissue expression (IHC) is inversely correlated with tumor size, TNM stage, and nuclear grade. In vitro blocked EMT via PI3K/Akt/GSK3 β/Snail	Potential IHC marker of tumor aggressiveness
	[37]	Frozen tumor and adjacent tissue (clear cell RCC). Preoperative blood serum	αKlotho is downregulated in tumor tissue at RNA (qRT-PCR) and protein (IHC) levels. Reduced serum levels (ELISA) associated with higher tumor volume, Fuhrman grade, clinical stage, reduced cancer-specific survival, and progression-free survival	Serum αKlotho levels using ELISA may predict prognosis, including survival.
Ovarian cancer	[38]	Tumor (high-grade papillary-serous adenocarcinoma) and adjacent ovarian tissues. 19 cancer cell lines	αKlotho was reduced in tumor versus adjacent tissues (IHC) and in 16/19 cell lines (qRT-PCR)	
	[43]	FFPE and frozen tumor and adjacent tissues. 7 cancer cell lines	αKlotho was reduced in tumor versus adjacent tissues (IHC). Reduction correlates with low survival. Tumor xenografts expressing αKlotho had a smaller size. *KLA^−/−^* mice showed higher IL-6 levels in response to xenografts	Tissue expression using IHC may predict survival
Acute myeloid leukemia	[39]	KG-1 cells	Exposure to miR-126-5p decreased αKlotho levels and induced Akt phosphorylation and cytarabine resistance	αKlotho may predict cytarabine resistance
Breast cancer	[21]	Frozen tumor and adjacent tissues. MDA-MB-231 and H357T cancer cell lines	αKlotho was downregulated in cancer versus adjacent tissue. Undetectable in both cell lines	
Follicular thyroid carcinoma	[40]	FTC133 and FTC238 cancer cell lines	αKlotho reduced cell proliferation and induced apoptosis in vitro	
Esophageal cancer	[41]	FFPE cancer and adjacent tissues. Blood serum from patients/controls	αKlotho was downregulated in cancer versus adjacent tissue (IHC). Correlates with improved survival inversely correlated with staging, grade, lymph node metastasis, and β-catenin. Serum levels are higher in patients versus controls	Tissue levels (IHC) may predict prognosis, including survival. Serum 327 pg/mL cut-off (ELISA) is diagnostic with a sensitivity of 81% and specificity of 81%
Colorectal cancer	[46]	FFPE tumor tissue. RKO and LoVo cancer cell lines, Wi-38, and HUVEC cells	Lower αKlotho (IHC) is associated with lower patient survival. αKlotho prevents pro-tumorigenic effects of senescent cells in vitro and in vivo via NFκB/CCL2 blockade	Tissue levels (IHC) may predict survival
	[48]	Six cancer cell lines and normal cells	FL-1 regulates αKlotho expression in cancer cells	
	[50]	CaCo-2 cells	αKlotho induces apoptosis via the TRAIL death receptor	
	[53]	HT29 cancer cell line, CCD841 cells	αKlotho induces apoptosis specifically in cancer cells	
Gastric cancer	[49]	6 cancer cell lines and normal cells	SOX17 regulates αKlotho expression in cancer cells in vitro	
	[52]	HGC-27, AGS, MKN-45, MGC-803, HE-293-T cancer cell lines, GES-1 cells	Circular RNA ITCH upregulates αKlotho by sponging out miR-199-5p, inhibiting cell proliferation, migration, invasion, and EMT	
Pancreatic cancer	[51]	TCGA pancreatic ductal adenocarcinoma datasets, 3 mouse models	Promoter methylation and mRNA downregulation are associated with reduced survival. αKlotho knockdown synergized with Kras mutation to promote carcinogenesis. Soluble αKlotho inhibited xenograft growth and promoted the survival of KPC mice	Methylation and expression levels may predict survival

**Table 3:** Studies dealing with βKlotho.

Cancer Type	Reference	Type of Sample	Main Findings	Potential Applications
Hepatocellular carcinoma	[26]	Tumor and adjacent tissue in Trizol	*KLB* gene expression is upregulated in cancer tissues. A >2-fold increase correlates with the development of multiple lesions.	Screening of patients who could benefit from anti-FGFR therapies. Prediction of lesion multiplicity.
	[54]	Cell lines and xenograft mouse model	βKlotho mediates FGF9 pro-survival functions via FGFR3 and FGFR4. Inhibiting βKlotho was more effective than inhibiting FGFR4.	Screening of patients who could benefit from anti-FGFR therapies.
Prostate cancer	[27]	Frozen primary tumor tissue, FFPE metastases. PC3, DU145, VCaP, LnCaP cancer cell lines, PNT1a cells	KLB gene expression observed with qRT-PCR in DU145 and VCaP only, and FGF19 showed stimulatory effects. βKlotho was detected in a majority of primary and metastatic lesions using IHC.	βKlotho IHC may be useful for screening patients who could benefit from anti-FGFR therapy.
Breast cancer	[21]	Frozen tumor and adjacent tissue. MDA-MB-231 and HS578T cancer cell lines	βKlotho was downregulated in cancer versus normal tissues and was undetectable in both cell lines, suggesting a tumor-suppressor role.	
Pancreatic adenocarcinoma	[44]	Gene expression data from the Gene Expression Omnibus database	High *KLB* mRNA expression is associated with increased overall survival.	*KLB* gene expression may be useful in predicting patient survival.

**Table 4:** Studies Dealing with γKlotho.

Cancer Type	Reference	Type of Sample	Main Findings	Potential Applications
Breast cancer	[21]	Frozen tumor and adjacent tissue. MDA-MB-231 and HS578T cancer cell lines.	*LCTL* gene expression is upregulated in cancer versus normal tissues, especially in triple-negative lesions, using qRT-PCR, correlating with increased cell proliferation, histological grade, TNM stage, and reduced progression-free survival.	*LCTL* gene expression using qRT-PCR may be useful in predicting patient survival.
Prostate	[47]	FFPE tumor tissue from castration-resistant prostate cancer and cell lines.	Higher γKlotho expression observed by IHC in tumor tissue correlates with reduced overall survival and poor response to docetaxel in patients and in a mouse xenograft model.	γKlotho IHC may predict overall survival and response to docetaxel in castration-resistant prostate cancer.
Bladder cancer	[22]	FFPE pre-treatment tumor tissue. UMUC3, MGH-U3 and J82 cells.	Higher γKlotho expression observed by IHC in muscle-invasive versus non-muscle-invasive lesions. In non-muscle-invasive lesions, γKlotho levels correlated with poor progression-free survival.	γKlotho IHC may predict overall survival in patients with non-muscle-invasive bladder cancer.

## Data Availability

The data produced in this study are available in this article.
